# A Complete Index to the Dental Cosmos

**Published:** 1884-12

**Authors:** 


### A Complete Index to the Dental Cosmos.

Compiled by JAMES E. DEXTER, M.D.S., at the instance of A. L. NORTHROP, D.D.S., M.D.S. Volumes 1 to 24 Inclusive.

This work, issued by the S. S. White Dental Manufacturing Co., is a very valuable one indeed, for any who wish to consult the Dental Cosmos—and who does not? It is a complete index of all the articles published in that journal, not only of the leading articles, but of even the smaller selections. The work is almost a necessary accompaniment of the Dental Cosmos, and it should be in the hands of every one who has a complete file of that journal; and to those who have not, the possession of this index would be a strong inducement to procure the whole file. The work is very complete and very valuable, and as to the style of its issue it is only necessary to bear in mind who published it.

